# Fluoroquinolone-Resistant Avian Pathogenic *Escherichia coli* Isolated from Asymptomatic Broiler Chickens in a Slaughterhouse in Northern Thailand

**DOI:** 10.3390/pathogens15030253

**Published:** 2026-02-27

**Authors:** Rapeepan Yongyod, Thanaporn Eiamsam-Ang, Narong Kamolrat, Sawita Srisawat, Hathaikan Walanan, Sumontha Chaisaeng, Kulsatri Sittichottumrong, Rujirat Hatrongjit, Terdsak Yano, Anusak Kerdsin

**Affiliations:** 1 Faculty of Public Health, Kasetsart University, Chalermphrakiat Sakon Nakhon Province Campus, Sakon Nakhon 47000, Thailand; rapeepan.y@ku.th (R.Y.); ss.ssawita@gmail.com (S.S.); sumonthachaisaeng@gmail.com (S.C.); kulsatri.si@ku.th (K.S.); 2 Faculty of Veterinary Medicine, Chiang Mai University, Chiang Mai 50100, Thailand; thanaporn.e@cmu.ac.th (T.E.-A.); hataikan79@gmail.com (H.W.); terdsak.yano@cmu.ac.th (T.Y.); 3 Faculty of Natural Resources and Agro-Industry, Kasetsart University, Chalermphrakiat Sakon Nakhon Province Campus, Sakon Nakhon 47000, Thailand; narong.ka@ku.th; 4 Department of General Science, Faculty of Science and Engineering, Kasetsart University, Chalermphrakiat Sakon Nakhon Campus, Sakon Nakhon 47000, Thailand; rujirat.ha@ku.th

**Keywords:** avian pathogenic *E. coli*, antimicrobial resistance, Clermont phylogroup, chicken, zoonotic risk

## Abstract

**Background**: Avian pathogenic *Escherichia coli* (APEC) are significant bacterial pathogens that cause economic losses in the poultry industry and can pose a potential foodborne zoonotic risk. Herein, we examined APEC distribution and antimicrobial resistance in *E. coli* isolated from slaughtered broiler chickens in northern Thailand. **Methods**: PCR was used to classify APEC as either virulent or avirulent on 108 stored *E. coli* strains, as well as to perform Clermont phylotyping. Antimicrobial susceptibility to ciprofloxacin, cefotaxime, ceftazidime, imipenem, and colistin was examined. **Results**: Of the 108 *E. coli* strains, 101 (93.5%) isolates were APEC, and the remaining isolates were non-APEC. Among the APEC isolates, 58.4% were classified as virulent APEC; these isolates showed a statistically significant association with phylogroups F and C and (*n* = 54, 56.8%) more frequently exhibited a ciprofloxacin-resistant phenotype than avirulent APEC (*n* = 35, 36.8%). Among the ten APEC virulence genes, *hlyF*, *iroC*, *iroN*, *iutA*, *O78*, and *ompT* were significantly associated with virulent APEC. **Conclusions**: This study reveals a high prevalence of virulent APEC with fluoroquinolone resistance in slaughtered broiler chickens. Almost all virulent APEC strains belong to phylogroups F and C. The prediction of virulent APEC using *hlyF*, *iroC*, *iroN*, *iutA*, *O78*, and *ompT* may be useful.

## 1. Introduction

*Escherichia coli* has been implicated in foodborne diseases associated with the consumption of contaminated food and water. It is a commensal inhabitant of the vertebrate gut, exhibits extensive genetic diversity, and has a wide geographical distribution. Generally, *E. coli* can be distinguished as commensal or pathogenic, with the latter further divided into intestinal pathogenic *E. coli* (InPEC) and extraintestinal pathogenic *E. coli* (ExPEC) [1]. ExPEC includes avian pathogenic *E. coli* (APEC), septicemia-associated *E. coli* (SEPEC), newborn meningitis *E. coli* (NMEC), uropathogenic *E. coli* (UPEC), mammary pathogenic *E. coli* (MPEC), and endometrial pathogenic *E. coli* (EnPEC) [1]. Among ExPEC, APEC causes avian colibacillosis, a range of severe systemic infections in poultry that includes septicemia, airsacculitis, perihepatitis, and peritonitis [2,3]. This disease syndrome is responsible for high morbidity, substantial mortality rates, and considerable production losses in the global poultry industry. APEC is also considered a significant public health threat to humans because of its potential foodborne zoonotic transmission, with poultry acting as an external reservoir for extraintestinal infections, including urinary tract infections (UTIs), septicemia, and meningitis [2,3].

Based on Clermont typing, *E. coli* is divided into eight major phylogroups (A, B1, B2, C, D, E, F, and G) and the cryptic *Escherichia* clades I to V, which differ in their phenotypic and genotypic characteristics, ecological niches, ability to cause disease, and carriage of antimicrobial-resistant (AMR) genes [4,5]. Phylogroups A, B2, and D are most commonly found in humans, whereas phylogroup B1 is prevalent in animals and the environment [6,7,8]. In addition, phylogroups B2, D, and F are mainly associated with extraintestinal pathogenic strains, whereas phylogroups A, B1, and C contain the most intestinal pathogenic *E. coli* strains [5]. *E. coli* phylogroup F is often associated with infections in both animals (especially chickens) and humans, particularly with extraintestinal pathogenic strains, and includes highly virulent and antimicrobial-resistant strains [5,9,10]. Phylogroup G, particularly sequence type complex 117, has been identified as highly virulent and antibiotic-resistant and is associated with poultry and human infections [4].

*E. coli* is one of the key targets for the monitoring and surveillance of sanitary indicators and AMR in humans, food and the food-supply chain, food-producing animals, fresh and processed meat, vegetables, drinking water, and the environment [11,12]. Therefore, monitoring AMR *E. coli* in food-producing animals, food, water, and the environment is essential for public health interventions. Among food-producing animals, broiler chicken is the most widely consumed meat globally, and its consumption is projected to continue rising [13,14]. AMR in chicken production is a growing concern because of the widespread use of antibiotics for disease prevention, treatment, and growth promotion [15,16]. This misuse can lead to the development of resistant bacteria in chickens, which can then be transmitted to humans through the food chain or through direct contact [15].

The global rise in AMR in *E. coli* in poultry—particularly extended-spectrum β-lactamase (ESBL)-producing and fluoroquinolone-resistant strains—represents a critical concern in both veterinary and human medicine [17,18,19,20,21]. Human and animal infections caused by these resistant phenotypes are now widely documented [22,23,24]. Despite extensive research on antimicrobial-resistant (AMR) *E. coli* in poultry in Southeast Asia, a critical knowledge gap remains regarding the convergence of ESBL production, fluoroquinolone resistance, and high-virulence genotypes in slaughtered broilers. While previous studies in Thailand have primarily focused on commensal *E. coli*, the specific risk posed by APEC-like strains entering the food chain remains poorly defined [25]. Furthermore, a recent study from eastern Thailand has documented a 100% multidrug-resistance (MDR) rate among ESBL-producing APEC, and high-risk ExPEC lineages have also been identified on retail carcasses [26]. However, the molecular typing and convergence of virulence and fluoroquinolone resistance at the slaughterhouse interface in Thailand remain poorly elucidated.

In this study, these knowledge gaps are addressed by integrating phenotypic resistance profiling with Clermont phylotyping and virulence gene detection, providing a comprehensive assessment of high-risk APEC at the slaughterhouse interface in northern Thailand. This is performed by characterizing *E. coli* isolates recovered from broiler cloacal samples obtained as part of a previous antimicrobial resistance (AMR) surveillance program at a slaughterhouse in northern Thailand. This research therefore elucidates the molecular convergence of resistance and virulence in poultry-derived *E. coli*. The findings offer essential data to inform and underscore the necessity of rigorous antimicrobial stewardship and zoonotic risk-mitigation efforts to protect both public health and veterinary interests.

## 2. Materials and Methods

### 2.1. E. coli Strains and Susceptibility

The stored *E. coli* strains (*n* = 108) used in this study were obtained from the antimicrobial resistance bacterial surveillance program conducted by the Faculty of Veterinary Medicine, Chiang Mai University, between March and April 2024. Pure cultures of *E. coli* strains were sent from the Faculty of Veterinary Medicine, Chiang Mai University, to the Faculty of Public Health, Kasetsart University, and stored at −80 °C until further analysis. The *E. coli* strains were subcultured on Rapid’ *E. coli* 2 medium (Bio-Rad, Hercules, CA, USA). DNA was extracted, and confirmation was performed using PCR as described elsewhere [27].

Antimicrobial susceptibility testing for cefotaxime, ceftazidime, imipenem, and ciprofloxacin was performed on the 108 *E. coli* strains using the disk diffusion assay according to CLSI guidelines [28]. Colistin susceptibility was determined using broth microdilution following the method described elsewhere [28]. Extended-spectrum β-lactamase (ESBL) production was analyzed using the double-disk assay according to CLSI guidelines [28].

### 2.2. Molecular Analysis

PCR detection of *bla*_CTX-M_ and carbapenemase genes was conducted as described elsewhere [25,29]. Because colistin-susceptible *E. coli* may carry mobile colistin-resistant genes (*mcr*) [30], multiplex PCR for *mcr-1* through *mcr-10* was performed using our routine assay. The assay was divided into two reactions: reaction I consisted of *mcr-1*, *mcr-4*, *mcr-5*, *mcr-6*, and *mcr-7*, while reaction II consisted of *mcr-2*, *mcr-3*, *mcr-8*, *mcr-9*, and *mcr-10*. The multiplex PCR reaction mixture (25 µL) used for the detection of *mcr* genes contained 1× JumpStart REDTaq ReadyMix (Sigma-Aldrich, Carlsbad, CA, USA) and the corresponding primers for each reaction (Table 1). The PCR conditions were as follows: initial activation of DNA polymerase at 95 °C for 3 min; 35 cycles of denaturation at 95 °C for 30 s; primer annealing and extension at 62 °C for 1.30 min; and a final extension at 72 °C for 5 min. A negative control was included in each run and consisted of the same reaction mixture with water instead of template DNA.

Clermont typing was performed via PCR as previously described [31,32]. APEC was identified using two schemes: (1) three virulence genes—*fimC*, *iucD*, and *papC* [33]—and (2) five virulence genes—*iroN*, *ompT*, *hlyF*, *iss*, and *iutA* [34]. In the case of APEC, pathogenic strains (hereafter referred to as virulent) and non-pathogenic strains (hereafter referred to as avirulent or avian fecal *E. coli* [AFEC]) were classified based on the study by Kazimierczak et al. [35], in which virulent APEC presented at least one of the genes *iroC*, *hlyF*, or *O78*, any dual combination of these genes, or all three genes, whereas avirulent (non-pathogenic) APEC lacked all three genes. Uropathogenic potential was classified based on the presence of the virulence marker genes *yfcV*, *fyuA*, or *chuA* [36].

The PCR products (5 µL) were analyzed using gel electrophoresis on 2% (*w*/*v*) agarose gel in 0.5× TBE buffer at a constant voltage of 100 V for 30 min (Mupid exU system; Takara, Tokyo, Japan). The gels were stained with ethidium bromide and visualized under ultraviolet light (GeneGenius Bioimaging System; SynGene, Cambridge, UK). The sizes of the PCR products were determined through a comparison with molecular-size standards (GeneRuler^TM^ 100 bp Plus DNA ladder; Thermo Fisher Scientific, Vilnius, Lithuania).

### 2.3. Statistical Analysis

Since this study utilized a cross-sectional design, Prevalence Ratios (PRs) were calculated to assess associations. PRs offer more precise and interpretable estimates of relative risk than odds ratios in cross-sectional settings, especially when prevalence is high. The analysis accounted for the independence of observations and confirmed the absence of multicollinearity between AMR phenotypes, Clermont phylogroups, and virulence markers. To account for variability and ensure the robustness of the estimates, 95% confidence intervals (CIs) were calculated using a robust variance estimator, which minimizes bias arising from potential heteroscedasticity in binomial data. PR, 95% confidence interval (95% CI), and *p*-values were determined using MedCalc Software (MedCalc Software Ltd., Ostend, Belgium; https://www.medcalc.org/en/calc/tests.php; Access date: 12 December 2025). A multivariable regression model was performed for 10 virulent markers using STATA program (Version 18.0, 2023) to adjust for phylogroups, ESBL status, and resistance to ciprofloxacin and cephalosporins, yielding adjusted prevalence ratios (aPR), corresponding 95% CI, and *p*-values. Statistical significance was defined as a two-tailed *p*-value < 0.05. *p*-values below this limit were considered to provide sufficient evidence against the null hypothesis.

## 3. Results

Among the 108 *E. coli* strains isolated from broiler chickens, 99% (*n* = 107) were successfully classified into Clermont phylogroups (Table 2). The predominant phylogroup was B1 (38.9%), followed by C (17.6%), F (13.9%), and A (10.2%). Of the 108 isolates, 96 (88.9%) were resistant to ciprofloxacin, and 16 (14.8%) were resistant to third-generation cephalosporins. Phylogroup B1 showed high resistance to ciprofloxacin (38.9%), cefotaxime (6.5%), and ceftazidime (6.5%), followed by phylogroup A. In contrast, phylogroups C, D, E, F, and the unidentified phylogroup demonstrated resistance to ciprofloxacin only. No strains were resistant to imipenem or colistin. ESBL production was detected in strains from phylogroups A and B1; however, no *bla*_CTX-M_ was detected. Carbapenemase genes (*bla*_KPC_, *bla*_NDM_, *bla*_IMP_, *bla*_VIM_, and *bla*_OXA-48-like_) and mobile colistin-resistant genes (*mcr-1* to *mcr-10*) were not detected in any of the 108 *E. coli* strains. Overall, high ciprofloxacin resistance (88.9%) was observed in almost all *E. coli* isolates, whereas resistance to third-generation cephalosporins (7.4%) and ESBL production (1.8%) was low (Table 2).

Determination of APEC among these 108 *E. coli* strains revealed that 101 (93.5%) isolates were APEC, whereas 7 isolates were non-APEC. Among the 101 APEC isolates, 59 (58.4%) and 42 (41.6%) were classified as virulent APEC and avirulent APEC (AFEC), respectively, based on the method described elsewhere [35]. As shown in Table 3, virulent APEC was most frequently distributed in phylogroups C, B1, and F, whereas avirulent APEC predominated in phylogroups B1 and A. When phylogroups F and B1 were compared, virulent APEC showed a statistically significant association with phylogroup F (PR = 1.71, 95% CI = 1.09–2.66, *p*-value = 0.0172). Similarly, virulent APEC was more significantly associated with phylogroup C than with phylogroup B1 (PR = 2.12, 95% CI = 1.46–3.06, *p*-value = 0.0001). When phylogroups C and F were combined, virulent APEC showed a stronger statistically significant association with these phylogroups than with phylogroups A and B1 (PR = 2.09, 95% CI = 1.45–3.02, *p*-value = 0.0001). In addition, 95 (87.9%) APEC isolates were not susceptible to ciprofloxacin (89 isolates were resistant). Virulent APEC (*n* = 54, 56.8%) more frequently exhibited a ciprofloxacin-resistant phenotype than avirulent APEC (*n* = 35, 36.8%). Both ESBL-producing strains in the current study belonged to virulent APEC.

The distribution of APEC marker genes in the 101 APEC isolates showed the following frequencies: 92.1% *fimC* (*n* = 93), 60.4% *ompT* (*n* = 61), 49.5% *hlyF* (*n* = 50), 44.5% *iutA* (*n* = 45), 39.6% *iss* (*n* = 40), 33.7% *iroC* (*n* = 34), 33.7% *iroN* (*n* = 34), 29.7% *iucD* (*n* = 30), 3.9% *O78* (*n* = 4), and 1.0% *papC* (*n* = 1). Among these marker genes, *hlyF*, *iroC*, *iroN*, *iutA*, *ompT*, and *O78* showed statistically significant association with virulent APEC based on adjusted prevalence ratio (Table 4). However, *iss*, *iucD*, *fimC*, and *papC* were not significantly associated with virulent APEC based on adjusted prevalence ratio.

We determined UPEC virulence marker genes in the 108 *E. coli* strains, specifically *yfcV*, *fyuA*, and *chuA*. We detected 22 (20.4%) virulent APEC isolates that carried UPEC marker genes, whereas 12 (11.1%) avirulent APEC isolates carried UPEC marker genes (Table 5). Virulent APEC in phylogroup F carried UPEC marker genes more frequently than avirulent APEC in other phylogroups, with a statistically significant association (PR = 2.92, 95% CI = 1.68–5.06, *p*-value = 0.0001). In addition, phylogroup F was more strongly associated with the presence of UPEC marker genes than the other phylogroups (PR = 3.16, 95% CI = 2.01–4.95, *p*-value < 0.0001).

## 4. Discussion

APEC is considered to have zoonotic potential because of foodborne transmission and its close genetic relationship to UPEC and NMEC [3]. Drug-resistant APEC strains can enter the food chain and have previously been isolated from retail chicken [3]. In this study, a few *E. coli* strains produced ESBL, whereas some strains were resistant to third-generation cephalosporins. This could be explained by the involvement of other resistance mechanisms, such as porin loss, efflux pump, biofilm formation, or other beta-lactamases [37]. Resistance to fluoroquinolones, a class of critically important antibiotics for human medicine, has been observed globally in APEC at high levels. The extensive use of fluoroquinolones in veterinary settings, particularly in densely populated poultry operations, has functioned as a powerful selective force and has led to the dominance of fluoroquinolone-resistant clones. In Korea, 46.2% of APEC strains have demonstrated resistance to both enrofloxacin and ciprofloxacin [38]. In Brazil, 31.2% and 47.7% of APEC isolates have been resistant to ciprofloxacin and norfloxacin, respectively [39]. In Nepal, fluoroquinolone-resistant APEC has been reported at very high levels (82.5% to ciprofloxacin and 92% to enrofloxacin) [40]. In the present study, 88.9% of *E. coli* isolates (*n* = 108) from broiler chickens were resistant to ciprofloxacin. Of these isolates, 94.4% of APEC were non-susceptible to ciprofloxacin, and 52.9% of pathogenic APEC were resistant to ciprofloxacin. The connection between fluoroquinolone-resistant APEC and human health is a critical component of the One Health framework. Restriction and stewardship of fluoroquinolone use on farms, mandatory biosecurity and environmental control, and continuous surveillance and intervention could help to reduce or mitigate the spread of fluoroquinolone-resistant APEC.

APEC strains usually possess at least five main virulence genes that are considered minimal predictors for APEC, including *iroN* (salmochelin siderophore receptor gene), *hlyF* (putative avian hemolysin), *iutA* (aerobactin siderophore receptor gene), *iss* (episomal enhanced serum survival gene), and *ompT* (outer membrane protease gene) [34,39]. In this study, *iroC*, *iroN*, *hlyF*, *iutA*, *O78*, and *ompT* showed statistically significant associations with specific virulent APEC strains, whereas the remaining four markers (*iss*, *iucD*, *papC*, and *fimC*) were not significantly associated with virulent APEC. While *iss* was associated with an adjusted prevalence ratio of 1.41, this association approached but did not reach statistical significance (*p* = 0.079). This marginal result may be due to a limited sample size, which likely resulted in insufficient statistical power to detect a significant effect. The prevalence of *iroC*, *iroN*, *hlyF*, *iutA*, *O78*, and *ompT* in our APEC strains was 33.7%, 33.7%, 49.5%, 44.5%, 3.9%, and 60.4%, respectively. By contrast, a previous study in Brazil has shown higher prevalence for *iroN* (84.9%), *hlyF* (89.7%), *iutA* (80.2%), and *ompT* (89.7%) [39]. Another study in Korea has revealed high prevalence of *iroN* (78.0%), *hlyF* (97.0%), and *ompT* (80.3%) in APEC strains [41]. A study conducted in Turkey has reported 63% *ompT*, 60% *iutA*, and 43% *hlyF* in APEC isolates [42]. The variability in the prevalence of these five genes may depend on the specific *E. coli* strain, phylogenetic group, isolation source, host, and geographic location [34,43,44]. A previous study has shown that *E. coli* isolates collected from healthy broilers and the environment have a prevalence of *iroN*, *hlyF*, *iutA*, *iss*, and *ompT* similar to that of APEC isolates collected from colibacillosis-afflicted broilers, indicating a greater risk of colibacillosis infection [45]. Similarly to our study, although all strains were collected from healthy poultry at the slaughterhouse, the virulent APEC detected suggests a high risk of causing infections in poultry and transmission to humans.

A previous study has demonstrated that three virulence marker genes, *iroC*, *hlyF*, and *O78*, can be applied to predict pathogenic (virulent) strains of APEC [35]. Using this scheme, we distinguished our APEC strains as virulent or pathogenic strains (58.4%) and avirulent or non-pathogenic strains (41.6%). This study has demonstrated that *hlyF*, *iroC*, and *iroN* showed high PRs with statistical significance for virulent APEC strains. Although the *O78* gene has been considered a marker for virulent APEC in a previous study [35], its prevalence was low in our analysis, despite being statistically significant. The *E. coli* serotypes associated with APEC disease include O1, O2, O5, O8, O9, O11, O15, O18, O21, O26, O33, O35–O36, O55, O78, O83, O86, O88, O100, O109, O111, O113–O115, O119, O124–O128, O142, O145, O153, O157, O166, O171, and O175, which vary according to geographic area, strain type, and pathogenicity [35,46]. This variation may indicate that APEC isolates in our study contain few *E. coli* strains of serotype O78 and may predominantly belong to other serotypes. A key limitation of this study, however, is that serotyping of the 101 APEC isolates was not performed; therefore, the serotypes of these *E. coli* stains remain unknown. Among the 10 virulence marker genes investigated, *hlyF*, *iroC*, *iroN*, *iutA*, *O78*, and *ompT* were useful predictors of APEC, with *hlyF*, *iroC*, and *iroN* serving as particularly strong predictors of virulent or pathogenic APEC strains.

Generally, phylogroups F, B2, and G are more frequently associated with APEC disease and high virulence. They have also often been linked to specific high-risk clonal groups and a rich repertoire of virulence genes [47,48,49]. Our study showed that APEC was mostly distributed in phylogroups F, B1, and C, and that phylogroups F and C were significantly associated with virulent APEC. A previous study revealed that APEC isolates belonged to four phylogenetic groups (A, B1, B2, and D), with phylogroups A and B1 being the most predominant [41]. One study reported that phylogroup G predominated among APEC from broiler clinical isolates, whereas phylogroups A and B1 were dominant among APEC from broiler gastrointestinal isolates. In contrast, phylogroup B2 was the most common among APEC from clinical isolates in Turkey [50]. In China, phylogroups A and B1 were found to be dominant in APEC isolates [51]. A study in Iran analyzed 72 APEC strains differentiated into seven phylogenetic groups (A, B1, B2, C, D, E, and F) and found that phylogroup D was the most prevalent [52]. Variations in the distribution of Clermont phylogroups among APEC isolates are often driven by geographic factors, specific clonal expansion, virulence potential, and AMR profiles. However, utilizing the Clermont phylotyping scheme in conjunction with key APEC virulence markers facilitates a more robust characterization of these isolates. This integrated approach helps to elucidate the clustering of strains with specific pathogenic potential, providing a clearer link between genetic lineage and clinical risk.

Despite the insights provided in this study, several limitations must be acknowledged. These include cross-sectional design, sample scope, and absence of genomic/serotype analysis. The primary constraint in this study is the lack of granular data regarding sampling sites, specific selection criteria, the number of slaughterhouses involved, and the number of avian subjects sampled. These data gaps preclude a comprehensive assessment of animal flow dynamics and precise epidemiological prevalence, thereby limiting the geographic generalizability and representativeness of the isolates. Consequently, while our findings provide a snapshot of broiler-specific isolates, their broader implications for public health cannot be fully elucidated without subsequent validation. Longitudinal research incorporating clinical human samples and expanded environmental surveillance is essential to define the zoonotic transmission pathways and determine the full scale of the public health risk. To address these gaps, future research should prioritize multi-sectoral surveillance that bridges the gap between veterinary and human medicine. Incorporating a ‘One Health’ approach—by including samples from environmental runoff, slaughterhouse wastewater, and human patients—would provide a more comprehensive understanding of the public health risk. Additionally, utilizing standardized sampling protocols across wider geographic regions will be essential in validating these findings and informing targeted intervention strategies. Nevertheless, this study identified a high prevalence of fluoroquinolone-resistant APEC, particularly highly virulent strains, among healthy slaughtered broilers. These strains represent a critical reservoir with the potential to cause systemic avian colibacillosis and pose a zoonotic risk to humans through direct exposure or foodborne transmission. To mitigate the dissemination of these resistant pathogens, a comprehensive One Health framework is thus essential. This must include heightened biosecurity and sanitation protocols across the production chain, rigorous antimicrobial stewardship at the farm level, robust laboratory surveillance, and the implementation of strategic vaccination programs.

## 5. Conclusions

This research highlights a concerning prevalence of virulent, fluoroquinolone-resistant APEC strains in slaughtered broilers, primarily within phylogroups F and C. These results demonstrate that rigorous antimicrobial stewardship and enhanced biosecurity protocols are essential to mitigate the dissemination of multidrug-resistant pathogens. Furthermore, the identified six virulence genes (*hlyF*, *iroC*, *iroN*, *iutA*, *O78*, and *ompT*) are highly predictive for early-warning surveillance within a One Health framework.

## Figures and Tables

**Table 1:** Primer sequences of *mcr* genes used in multiplex PCR.

Primer Name	Sequence (5′–3′)	Reaction	Product Size (bp)	Reference
mcr1-F	TTG CCA AAT TCA CGC CAG TG	1	384	This study
mcr1-R	CTT TGA CTT TGT CCG CGG TG			
mcr2-F	CAA GTG TGT TGG TCG CAG TT	2	715	[30]
mcr2-R	TCT AGC CCG ACA AGC ATA CC			
mcr3-F	CTG AAC TGG CGT GGA GTT CT	2	1375	This study
mcr3-R	ATC ATC CGG TGC AAA CTG GT			
mcr4-F	TCA CTT TCA TCA CTG CGT TG	1	1100	This study
mcr4-R	TTG GTC CAT GAC TAC CAA TG			
mcr5-F	ATG CGG TTG TCT GCA TTT ATC	1	1644	[30]
mcr5-R	TCA TTG TGG TTG TCC TTT TCT G			
mcr6-F	GTC CGG TCA ATC CCT ATC TGT	1	556	[30]
mcr6-R	ATC ACG GGA TTG ACA TAG CTA C			
mcr7-F	TGC TCA AGC CCT TCT TTT CGT	1	892	[30]
mcr7-R	TTC ATC TGC GCC ACC TCG T			
mcr8-F	AAC CGC CAG AGC ACA GAA TT	2	667	[30]
mcr8-R	TTC CCC CAG CGA TTC TCC AT			
mcr9-F	CTT TCC ATA ACA GCG AGA CAC	2	573	[30]
mcr9-R	GTA TCC TTC CTG CCA TCC TC			
mcr10-F	CGCTTCGCTGATCCTGATTG	2	901	This study
mcr10-R	GCTCTCTCCCAGAGATTCGC			

**Table 2:** *E. coli* phylogroups (*n* = 108), antimicrobial susceptibility, and antimicrobial-resistant genes.

Phylogroups	Resistance to					ESBL- Production	Antimicrobial-Resistant Gene		
	CIP (%)	CTX (%)	CAZ (%)	IMP (%)	COL (%)		*bla*_CTX-M_	* Carbapenemase	*mcr-1*– *mcr-10*
A	11 (10.2)	1 (0.9)	1 (0.9)	0	0	1 (0.9)	0	0	0
B1	42 (38.9)	7 (6.5)	7 (6.5)	0	0	1 (0.9)	0	0	0
C	19 (17.6)	0	0	0	0	0	0	0	0
D	4 (3.7)	0	0	0	0	0	0	0	0
E	4 (3.7)	0	0	0	0	0	0	0	0
F	15 (13.9)	0	0	0	0	0	0	0	0
Unidentified	1 (0.9)	0	0	0	0	0	0	0	0
Total	96 (88.9)	8 (7.4)	8 (7.4)	0	0	2 (1.8)	0	0	0

CIP = ciprofloxacin, CTX = cefotaxime, CAZ = ceftazidime, IMP = imipenem, COL = colistin. * Carbapenemase genes: *bla*_KPC_, *bla*_NDM_, *bla*_IMP_, *bla*_VIM_, and *bla*_OXA-48-like_.

**Table 3:** Distribution of *E. coli* phylogroups and APEC types.

APEC Type	Phylogroup							
	A	B1	C	D	E	F	Unknown	Total
Virulent APEC	4	17	18	3	4	13		59
Avirulent APEC	12	21	1	3		4	1	42
Non-APEC	2	5						7
**Total**	**18**	**43**	**19**	**6**	**4**	**17**	**1**	**108**

**Table 4:** Statistical analysis of APEC marker genes in 101 APEC isolates.

APEC Marker Gene	Crude Prevalence Ratio (95% CI)	*p*-Value	Adjusted Prevalence Ratio (95%CI)	*p*-Value
*hlyF*	72.38 (4.59–1141.22)	0.0023	5.37 (2.81–10.26)	<0.001
*iroC*	49.45 (3.12–784.64)	0.0057	2.55 (1.79–3.63)	<0.001
*iroN*	49.45 (3.12–784.64)	0.0057	2.55 (1.79–3.63)	<0.001
*iutA*	3.20 (1.66–6.17)	0.0005	1.83 (1.23–2.71)	0.003
*iss*	2.45 (1.31–4.59)	0.0051	1.41 (0.96–2.05)	0.079
*iucD*	2.34 (1.11–4.94)	0.0259	1.34 (0.91–1.96)	0.140
*ompT*	2.18 (1.42–3.35)	0.0003	2.14 (1.16–3.98)	0.015
*fimC*	0.90 (0.81–1.00)	0.0561	0.90 (0.50–1.61)	0.728
*papC*	0.24 (0.01–5.72)	0.3777	0.12 (0.01–3.22)	0.1271
*O78*	6.45 (0.35–116.68)	0.2070	3.34 (2.05–5.44)	<0.001

**Table 5:** Distribution of *E. coli* phylogroups, types of APEC, and UPEC marker genes.

APEC Type	Phylogroup							
	A	B1	C	D	E	F	Unknown	Total
**Virulent APEC (*n* = 59)**								
- Presence of UPEC marker genes		5	2	1	3	11		**22**
- Absence of UPEC marker genes	4	12	16	2	1	2		**37**
**Avirulent APEC (*n* = 42)**								
- Presence of UPEC marker genes	5	4	1	1		2		**13**
- Absence of UPEC marker genes	7	17		2		2	1	**29**
**Non-APEC (*n* = 7)**								
- Presence of UPEC marker genes								
- Absence of UPEC marker genes	2	5						**7**
**Total**	**18**	**43**	**19**	**6**	**4**	**17**	**1**	**108**

## Data Availability

The original contributions presented in this study are included in the article. Further inquiries can be directed to the corresponding author.

## Abbreviations

The following abbreviations are used in this manuscript:

AMR	Antimicrobial resistance
PCR	Polymerase chain reaction
ESBL	Extended-spectrum β-lactamase
ExPEC	Extraintestinal pathogenic *Escharichia coli*
UPEC	Uropathogenic *Escharichia coli*
APEC	Avian pathogenic *Escharichia coli*

## References

[1] Alhadlaq M.A., Aljurayyad O.I., Almansour A., Al-Akeel S.I., Alzahrani K.O., Alsalman S.A., Yahya R., Al-Hindi R.R., Hakami M.A., Alshahrani S.D. (2024). Overview of pathogenic *Escherichia coli*, with a focus on Shiga toxin-producing serotypes, global outbreaks (1982–2024) and food safety criteria. Gut Pathog..

[2] Kathayat D., Lokesh D., Ranjit S., Rajashekara G. (2021). Avian Pathogenic *Escherichia coli* (APEC): An Overview of Virulence and Pathogenesis Factors, Zoonotic Potential, and Control Strategies. Pathogens.

[3] Watts A., Wigley P. (2024). Avian Pathogenic *Escherichia coli*: An Overview of Infection Biology, Antimicrobial Resistance and Vaccination. Antibiotics.

[4] Clermont O., Dixit O.V.A., Vangchhia B., Condamine B., Dion S., Bridier-Nahmias A., Denamur E., Gordon D. (2019). Characterization and rapid identification of phylogroup G in *Escherichia coli*, a lineage with high virulence and antibiotic resistance potential. Environ. Microbiol..

[5] Lagerstrom K.M., Hadly E.A. (2023). Under-Appreciated Phylogroup Diversity of *Escherichia coli* within and between Animals at the Urban-Wildland Interface. Appl. Environ. Microbiol..

[6] Tenaillon O., Skurnik D., Picard B., Denamur E. (2010). The population genetics of commensal *Escherichia coli*. Nat. Rev. Microbiol..

[7] Smati M., Clermont O., Le Gal F., Schichmanoff O., Jauréguy F., Eddi A., Denamur E., Picard B. (2013). Real-time PCR for quantitative analysis of human commensal *Escherichia coli* populations reveals a high frequency of subdominant phylogroups. Appl. Environ. Microbiol..

[8] Méric G., Kemsley E.K., Falush D., Saggers E.J., Lucchini S. (2013). Phylogenetic distribution of traits associated with plant colonization in *Escherichia coli*. Environ. Microbiol..

[9] Zhuge X., Zhou Z., Jiang M., Wang Z., Sun Y., Tang F., Xue F., Ren J., Dai J. (2021). Chicken-source *Escherichia coli* within phylogroup F shares virulence genotypes and is closely related to extraintestinal pathogenic *E. coli* causing human infections. Transbound. Emerg. Dis..

[10] Vangchhia B., Abraham S., Bell J.M., Collignon P., Gibson J.S., Ingram P.R., Johnson J.R., Kennedy K., Trott D.J., Turnidge J.D. (2016). Phylogenetic diversity, antimicrobial susceptibility and virulence characteristics of phylogroup F *Escherichia coli* in Australia. Microbiology.

[11] Food and Agriculture Organization (2019). Monitoring and surveillance of antimicrobial resistance in bacteria from healthy food animals intended for consumption. Regional Antimicrobial Resistance Monitoring and Surveillance Guidelines—Volume 1.

[12] World Health Organization (2017). Integrated Surveillance of Antimicrobial Resistance in Foodborne Bacteria: Application of a One Health Approach.

[13] Mottet A., Tempio G. (2017). Global poultry production: Current state and future outlook and challenges. World’s Poult. Sci. J..

[14] Speedy A.W. (2003). Global production and consumption of animal source foods. J. Nutr..

[15] Roth N., Käsbohrer A., Mayrhofer S., Zitz U., Hofacre C., Domig K.J. (2019). The application of antibiotics in broiler production and the resulting antibiotic resistance in *Escherichia coli*: A global overview. Poult. Sci..

[16] Ardakani Z., Aragrande M., Canali M. (2024). Global antimicrobial use in livestock farming: An estimate for cattle, chickens, and pigs. Animal.

[17] Misumi W., Magome A., Okuhama E., Uchimura E., Tamamura-Andoh Y., Watanabe Y., Kusumoto M. (2023). CTX-M-55-type ESBL-producing fluoroquinolone-resistant *Escherichia coli* sequence type 23 repeatedly caused avian colibacillosis in Kagoshima Prefecture, Japan. J. Glob. Antimicrob. Resist..

[18] Park H., Kim J., Ryu S., Jeon B. (2022). The rate of frequent co-existence of plasmid-mediated quinolone resistance (PMQR) and extended-spectrum β-lactamase (ESBL) genes in *Escherichia coli* isolates from retail raw chicken in South Korea. Food Sci. Biotechnol..

[19] Das T., Nath C., Das P., Ghosh K., Logno T.A., Debnath P., Dash S., Devnath H.S., Das S., Islam M.Z. (2023). High prevalence of ciprofloxacin resistance in *Escherichia coli* isolated from chickens, humans and the environment: An emerging one health issue. PLoS ONE.

[20] De Koster S., Ringenier M., Lammens C., Stegeman A., Tobias T., Velkers F., Vernooij H., Kluytmans-van den Bergh M., Kluytmans J., Dewulf J. (2021). ESBL-Producing, Carbapenem- and Ciprofloxacin-Resistant *Escherichia coli* in Belgian and Dutch Broiler and Pig Farms: A Cross-Sectional and Cross-Border Study. Antibiotics.

[21] De Koster S., Ringenier M., Xavier B.B., Lammens C., De Coninck D., De Bruyne K., Mensaert K., Kluytmans-van den Bergh M., Kluytmans J., Dewulf J. (2023). Genetic characterization of ESBL-producing and ciprofloxacin-resistant *Escherichia coli* from Belgian broilers and pigs. Front. Microbiol..

[22] Husna A., Rahman M.M., Badruzzaman A.T.M., Sikder M.H., Islam M.R., Rahman M.T., Alam J., Ashour H.M. (2023). Extended-spectrum β-lactamases (ESBL): Challenges and opportunities. Biomedicines.

[23] Boueroy P., Chopjitt P., Hatrongjit R., Morita M., Sugawara Y., Akeda Y., Iida T., Hamada S., Kerdsin A. (2023). Fluoroquinolone resistance determinants in carbapenem-resistant *Escherichia coli* isolated from urine clinical samples in Thailand. PeerJ.

[24] Belas A., Marques C., Menezes J., da Gama L.T., Cavaco-Silva P., Pomba C. (2022). ESBL/pAmpC-Producing *Escherichia coli* Causing Urinary Tract Infections in Non-Related Companion Animals and Humans. Antibiotics.

[25] Laopiem S., Witoonsatian K., Kulprasetsri S., Panomwan P., Pathomchai-Umporn C., Kamtae R., Jirawattanapong P., Songserm T., Sinwat N. (2025). Antimicrobial resistance, virulence gene profiles, and phylogenetic groups of *Escherichia coli* isolated from healthy broilers and broilers with colibacillosis in Thailand. BMC Vet. Res..

[26] Tongkamsai S., Nakbubpa K. (2023). Extended-spectrum beta-lactamase (ESBL) production and virulence genes profile of avian pathogenic *Escherichia coli* (APEC) isolated from broiler chickens in eastern Thailand. Vet. Integr. Sci..

[27] Hatrongjit R., Chaisaeng S., Sitthichotthumrong K., Boueroy P., Chopjitt P., Ungcharoen R., Kerdsin A. (2025). Polymerase Chain Reaction-Lateral Flow Strip for Detecting *Escherichia coli* and *Salmonella enterica* Harboring blaCTX-M. Antibiotics.

[28] (2025). Performance Standards for Antimicrobial Susceptibility Testing.

[29] Hatrongjit R., Chopjitt P., Boueroy P., Kerdsin A. (2022). Multiplex PCR Detection of Common Carbapenemase Genes and Identification of Clinically Relevant *Escherichia coli* and *Klebsiella pneumoniae* Complex. Antibiotics.

[30] Khanawapee A., Kerdsin A., Chopjitt P., Boueroy P., Hatrongjit R., Akeda Y., Tomono K., Nuanualsuwan S., Hamada S. (2021). Distribution and Molecular Characterization of *Escherichia coli* Harboring *mcr* Genes Isolated from Slaughtered Pigs in Thailand. Microb. Drug Resist..

[31] Clermont O., Gordon D.M., Brisse S., Walk S.T., Denamur E. (2011). Characterization of the cryptic *Escherichia lineages*: Rapid identification and prevalence. Environ. Microbiol..

[32] Clermont O., Christenson J.K., Denamur E., Gordon D.M. (2013). The Clermont *Escherichia coli* phylo-typing method revisited: Improvement of specificity and detection of new phylo-groups. Environ. Microbiol. Rep..

[33] Ievy S., Islam M.S., Sobur M.A., Talukder M., Rahman M.B., Khan M.F.R., Rahman M.T. (2020). Molecular Detection of Avian Pathogenic *Escherichia coli* (APEC) for the First Time in Layer Farms in Bangladesh and Their Antibiotic Resistance Patterns. Microorganisms.

[34] Johnson T.J., Wannemuehler Y., Doetkott C., Johnson S.J., Rosenberger S.C., Nolan L.K. (2008). Identification of minimal predictors of avian pathogenic *Escherichia coli* virulence for use as a rapid diagnostic tool. J. Clin. Microbiol..

[35] Kazimierczak J., Pospiech K., Sowińska P., Pękala A., Borówka P., Wójcik E.A., Marciniak B., Lis M.W., Strapagiel D., Dastych J. (2025). A rapid detection of Avian Pathogenic *Escherichia coli* (APEC) strains based on minimal number of virulence markers identified by whole genome sequencing. BMC Microbiol..

[36] Spurbeck R.R., Dinh P.C., Walk S.T., Stapleton A.E., Hooton T.M., Nolan L.K., Kim K.S., Johnson J.R., Mobley H.L. (2012). *Escherichia coli* isolates that carry vat, fyuA, chuA, and yfcV efficiently colonize the urinary tract. Infect. Immun..

[37] Nasrollahian S., Graham J.P., Halaji M. (2024). A review of the mechanisms that confer antibiotic resistance in pathotypes of *E. coli*. Front. Cell. Infect. Microbiol..

[38] Yoon M.Y., Kim Y.B., Ha J.S., Seo K.W., Noh E.B., Son S.H., Lee Y.J. (2020). Molecular characteristics of fluoroquinolone-resistant avian pathogenic *Escherichia coli* isolated from broiler chickens. Poult. Sci..

[39] Lúcio C.J., Hansen P.H.C., Griebeler J., Kipper D., Lunge V.R. (2025). Virulence and Antimicrobial Resistance of Avian Pathogenic *Escherichia coli* (APEC) Isolates from Poultry in Brazil. Poultry.

[40] Bhattarai R.K., Basnet H.B., Dhakal I.P., Devkota B. (2024). Antimicrobial resistance of avian pathogenic *Escherichia coli* isolated from broiler, layer, and breeder chickens. Vet. World.

[41] Kim J.H., Lee H.J., Jeong O.M., Kim D.W., Jeong J.Y., Kwon Y.K., Kang M.S. (2021). High prevalence and variable fitness of fluoroquinolone-resistant avian pathogenic *Escherichia coli* isolated from chickens in Korea. Avian Pathol..

[42] Sariçam İnce S., Ünal A., Akan M. (2025). Comparison of pathogenicity factors of avian pathogenic and extraintestinal pathogenic *Escherichia coli* isolates originating from broiler chickens. Br. Poult. Sci..

[43] Ovi F., Zhang L., Nabors H., Jia L., Adhikari P. (2023). A compilation of virulence-associated genes that are frequently reported in avian pathogenic *Escherichia coli* (APEC) compared to other *E. coli*. J. Appl. Microbiol..

[44] Sarowska J., Olszak T., Jama-Kmiecik A., Frej-Madrzak M., Futoma-Koloch B., Gawel A., Drulis-Kawa Z., Choroszy-Krol I. (2022). Comparative Characteristics and Pathogenic Potential of *Escherichia coli* Isolates Originating from Poultry Farms, Retail Meat, and Human Urinary Tract Infection. Life.

[45] Fancher C.A., Thames H.T., Colvin M.G., Smith M., Easterling A., Nuthalapati N., Zhang L., Kiess A., Dinh T.T.N., Theradiyil Sukumaran A. (2021). Prevalence and Molecular Characteristics of Avian Pathogenic *Escherichia coli* in “No Antibiotics Ever” Broiler Farms. Microbiol. Spectr..

[46] Nawaz S., Wang Z., Zhang Y., Jia Y., Jiang W., Chen Z., Yin H., Huang C., Han X. (2024). Avian pathogenic *Escherichia coli* (APEC): Current insights and future challenges. Poult. Sci..

[47] Adams J.R.G., Long H., Birdsall C.A., Qureshi K., King E., Perez S., Warner K., Behboudi S., La Ragione R.M., Mehat J.W. (2025). High-risk clonal groups of avian pathogenic *Escherichia coli* (APEC) demonstrate heterogeneous phenotypic characteristics in vitro and in vivo. Virulence.

[48] Murase T., Ozaki H. (2022). Relationship between phylogenetic groups of *Escherichia coli* and Pathogenicity among Isolates from chickens with Colibacillosis and healthy chickens. Poult. Sci..

[49] Cordoni G., Woodward M.J., Wu H., Alanazi M., Wallis T., La Ragione R.M. (2016). Comparative genomics of European avian pathogenic *E. coli* (APEC). BMC Genom..

[50] Delago J., Miller E.A., Flores-Figueroa C., Munoz-Aguayo J., Cardona C., Smith A.H., Johnson T.J. (2023). Survey of clinical and commensal *Escherichia coli* from commercial broilers and turkeys, with emphasis on high-risk clones using APECTyper. Poult. Sci..

[51] Song S., Wang Y., Liu Z., Zhang R., Li K., Yin B., Yan Z., Yang S., Lin S., Yi Y. (2025). Population Structure, Genomic Features, and Antibiotic Resistance of Avian Pathogenic *Escherichia coli* in Shandong Province and Adjacent Regions, China (2008–2023). Microorganisms.

[52] Tohmaz M., Askari Badouei M., Kalateh Rahmani H., Hashemi Tabar G. (2022). Antimicrobial resistance, virulence-associated genes and phylogenetic background versus plasmid replicon types: The possible associations in avian pathogenic *Escherichia coli* (APEC). BMC Vet. Res..

